# Influence of smoking and smoking cessation on levels of urinary 11-dehydro thromboxane B_2_

**DOI:** 10.1016/j.toxrep.2018.04.005

**Published:** 2018-04-19

**Authors:** Angela van der Plas, Sandrine Pouly, Guillaume de La Bourdonnaye, Wee Teck Ng, Gizelle Baker, Frank Lüdicke

**Affiliations:** Product Assessment and Scientific Substantiation, Philip Morris International Research & Development, Philip Morris Products S.A., Quai Jeanrenaud 5, 2000 Neuchatel, Switzerland

**Keywords:** Smoking, Inflammation, Thromboxane, Meta-analysis

## Abstract

**Background:**

Thromboxane is a key clinical risk endpoint of smoking-induced inflammation which has been associated in the pathogenesis of cardiovascular disease. The goal of this review is to quantify the effect of smoking and smoking cessation on one of its urinary metabolites, 11-dehydrothromboxane_B2_.

**Methods:**

PubMed and SCOPUS were searched to identify publications which report urinary 11-dehydrothromboxane_B2_ levels in smokers and non-smokers, as well as articles reporting the effect of smoking cessation on urinary 11-dehydrothromboxane_B2_ excretion.

**Results:**

We found ten studies assessing urinary 11-dehydrothroboxane_B2_ levels in smokers and non-smokers. Four papers reported the amount of urinary 11-dehydrothromboxane_B2_ excreted in 24 h while six reported the amount excreted adjusted for creatinine. The meta-analyses comparing the excretion of urinary 11-dehydrothromboxane in current smokers to non-smokers report increased levels in current smokers (mean difference = 0.31 μg/24-h [95%CI: 0.27–0.34] and 166.45 pg/mg creatinine [95%CI: 120.51–212.40]). There were not enough publications to perform meta-analyses on the effects of smoking cessation on urinary 11-dehydrothromboxane_B2_ excretion.

**Conclusions:**

Urinary 11-dehydrothromboxane_B2_ levels are increased in cigarette smokers, however, more data are needed to elucidate the effects of smoking cessation on urinary 11-dehydrothromboxane_B2_ excretion.

## 1 Introduction

Cigarette smoking is an important modifiable risk factors for cardiovascular diseases (CVD) such as myocardial infarction, sudden death and stroke [[1], [2], [3]]. For instance, women smokers of 25 or more cigarettes per day have a relative risk (RR) of 5.4 (95% CI: 3.0–10.4) for fatal coronary heart disease (CHD) and 5.8 (95% CI: 4.2-8.0) for nonfatal myocardial infarction in comparison to non-smokers [4] while in men, the RR for myocardial infarction (fatal and non-fatal) for smokers *vs*. non-smokers is 3.63 (95%CI: 3.03-4.35) [5]. After smoking cessation, the risks for cerebrovascular and ischemic heart disease reduce by 50% after 4.78 years (95%CI: 2.17–10.50) [6] and 4.40 years (95%CI: 3.26–5.95) [7] respectively.

Alternatives to cigarettes are being developed and marketed. These alternatives deliver nicotine but reduce the exposure to harmful chemicals and therefore have the potential to reduce the risk of smoking related diseases compared to continued smoking. As these products become available, it will be important to provide consumers accurate information about the potential risk reduction. In the absence of long term epidemiological studies, the evaluation of risk modification through the use of products substituting combustible tobacco products may not be timely enough to address the public health opportunity these new products may offer. Thus, the study of clinical risk endpoints has become an integral component of PMI’s assessment of how the reduction of toxicants in the inhaled aerosol by the consumer translates into a proxy of smoking-related disease.

Candidate endpoints of risk should be involved in biological pathways known to be affected by smoking, such as the in inflammatory response or plaque formation on arterial walls [8,9]. One of the biomarkers highlighted in the CVD and smoking-related disease literature is thromboxane, which is reported as a mediator involved in the pathogenesis of cardiovascular diseases [10]. Smoking has been associated with enhanced thromboxane A2 release by platelets in healthy individuals [11] and several studies have assessed the levels of thromboxane A2 in the plasma of smokers compared to non-smokers [12,13]. As well, the excretion of the two major urinary metabolites of thromboxane A2, namely 2,3-dinor-thromboxane_B2_ [1,14] and 11-dehydro-thromboxane_B2_ (TXB2)^1^ [1] have been studied. Researchers have also assessed the effect of smoking cessation [15] on urinary TXB2 levels, where the data show that as early as three days after smoking cessation (without nicotine substitution), the TXB2 levels were lowered to levels of about 75% (p-value < 0.01) of the baseline values, and after 14 days, the levels were reduced to 50% (p-value < 0.01) of the baseline values.

The aim of this research was to assess the association of smoking status and urinary levels of TXB2 by reviewing and analysing the published available literature on: a) urinary TXB2 levels in smokers *vs*. non-smokers and b) the influence of smoking cessation on urinary TXB2 levels.

## 2 Methods

Medline searches were performed through PubMed and additionally in the SCOPUS database, for publications that evaluated the relationship between smoking or smoking cessation and urinary TXB2 levels. The final search was performed on March 9th 2018. The following query was used in PubMed: ("thromboxanes"[MeSH Terms] OR "thromboxanes"[All Fields] OR "thromboxane"[All Fields]) AND (("smoking"[MeSH Terms] OR "smoking"[All Fields]) OR ("tobacco"[MeSH Terms] OR "tobacco"[All Fields] OR "tobacco products"[MeSH Terms] OR ("tobacco"[All Fields] AND "products"[All Fields]) OR "tobacco products"[All Fields]) OR cessation[All Fields] OR quitting[All Fields]). In SCOPUS the following query was used: Thromboxane AND (smoking OR tobacco OR cessation OR quitting).

Retrieval of articles was limited to those written in English and considering human populations. To verify that all available publications were retrieved, the reference lists of the publications obtained through the original search were reviewed to identify any additional citation.

### 2.1 Study selection

The following criteria were used for including/excluding publications from the review:

a) Inclusion Criteria:
b) Case control, cross-sectional, cohort or interventional studies such as randomized controlled trials
c) Adult healthy human populations
d) Measurements of TXB2 by exposure with the following measures available: mean values by group, standard deviation (SD) or standard error (SE) (of the mean), sample size per group or with enough information to allow for the calculation of mean and SD
e) Studies published from 1970 until March 9th 2018
f) Exclusion Criteria:
g) Review articles, case reports or editorials
h) Studies with incomplete data
i) Studies where data had been re-used in a more recent study
j) Studies including diseased populations

### 2.2 Data extraction

Two researchers extracted data independently, discussed any disagreements and reached consensus on all items. The following information was extracted from each study: the first author’s name, year of publication, study design and population characteristics, number of participants per group, mean, standard deviation (SD) or standard error (SE). Not all articles reported the measurements in the same units, so only publications where the values could be transformed to either pg/mg or μg/24-h were used. Transformation from median and range values was performed according to the calculations postulated by Hozo et al. [16].

### 2.3 Statistical analysis

Pooled means levels of urinary TXB2 by exposure group (smokers and non-smokers) were calculated by weighting the individual studies by their inverse pooled variance. To quantify the effects of smoking and smoking cessation on TXB2, pooled mean differences between smokers and non-smokers and 95% confidence intervals (95% CIs) were calculated using the fixed-effects model in Review Manager version 5.0 (Cochrane Collaboration, Oxford, UK). The degree of heterogeneity between the study results was tested by the inconsistency statistic (I^2^). Funnel plots were used to evaluate publication bias [17]. Statistical significance was assessed at α = 0.05.

## 3 Results

**Fig. 1:**
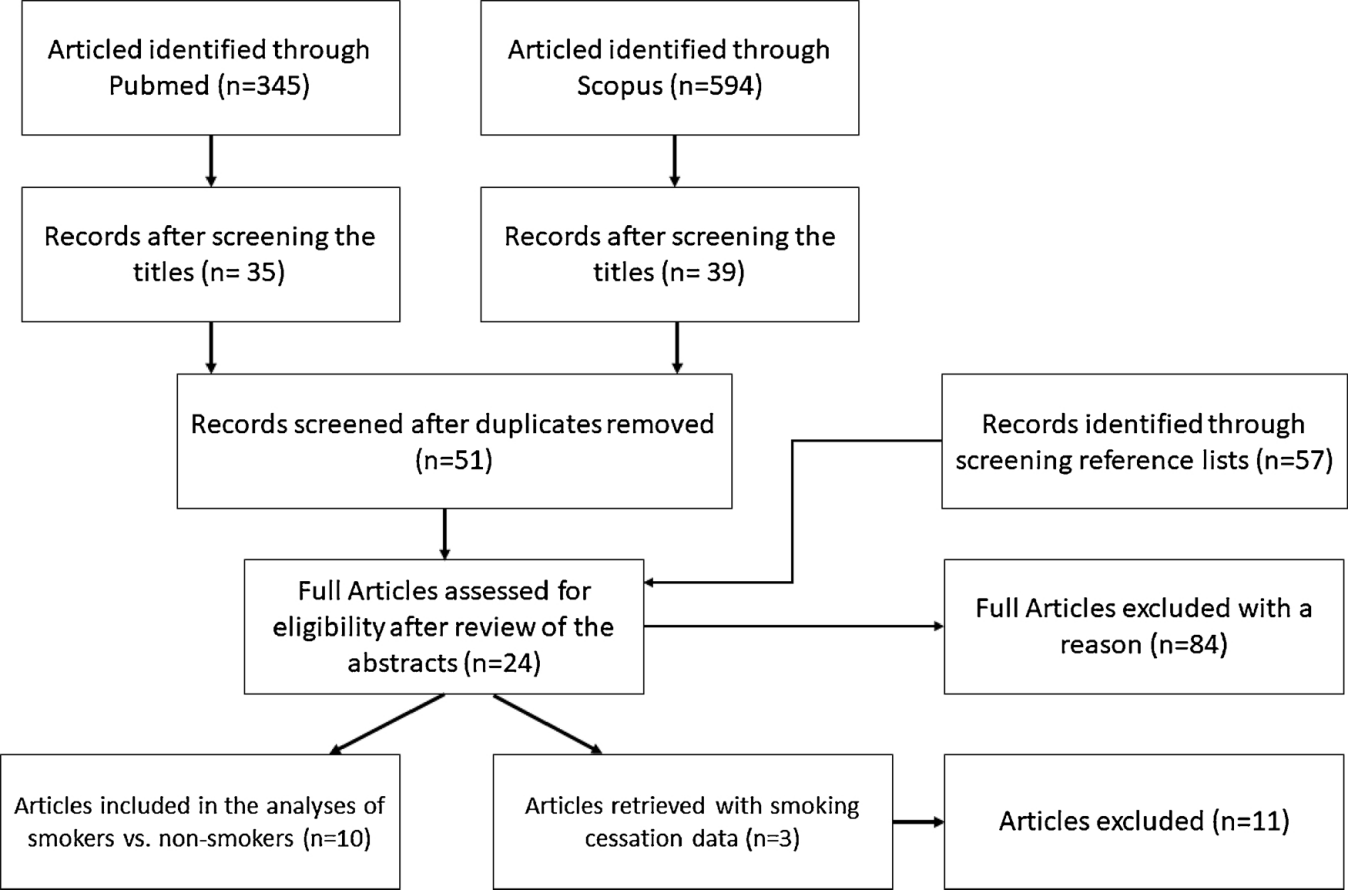
Flow diagram – article retrieval process.

A flow diagram detailing the article retrieval process from the different sources used can be found in Fig. 1. For the analyses of urinary TXB2 levels and its association to smoking status, a total of 21 studies were identified where the levels of urinary TXB2 were compared in smokers *vs*. non-smokers. Of the 21 studies, ten were included in the meta-analyses [10,14,[18], [19], [20], [21], [22], [23], [24], [25]]. The other 11 studies were not included due to having incomplete information [26,27], results given in different units [[28], [29], [30], [31]], study results had been published in another study [1,32], measurements were reported from plasma concentrations [13] or included diseased populations [33,34]. Characteristics of all studies included in the analyses can be found in Table 1. The included studies reported one of two kind of measurements of urinary TXB2, namely, six reported spot urine or 24-h urine TXB2 concentration corrected for creatinine concentration [10,19,[22], [23], [24],^35^] whilst four reported the total urinary excretion of TXB2 over 24-h [14,18,36]. For the analysis of the effect of smoking cessation on urinary TXB2 levels, three studies [15,26,37] were identified, but no meta-analysis could performed because the studies had different follow-up duration, ranging from 3 days to 14 days Their characteristics can be found in Table 2.

**Table 1:** Characteristics of studies assessing levels of TXB2 in Smokers *vs*. Non-Smokers.

Reference	Country	Study Design	Study participants	Smoking Definition	Subgroup			Units	Adjustment
		8			Smokers	Non-smokers	Mean Difference		
					Mean ± SD	Mean ± SD	Δ (95%CI)		
Barrow et al. [19]	UK	Cross sectional	67 males aged 18–39 years	None	All 440 ± 295.8	221 ± 109.49	219 (93.27, 344.73)	pg/mg creatinine	None
Uedelhoven et al. [23]	Germany	Cross sectional	23 healthy men and women aged 26–56 years	None	All 673.2 ± 320.5	332.6 ± 97.7	340.60 (156.15, 525.05)	pg/mg creatinine	None
Uyama et al. [25]	Japan	Cross sectional	44 male and female healthy participants aged 31–80 years	5 CPD+	All 1063 ± 244	815 ± 183*	248 (113.03, 382.97)	pg/mg creatinine	None
McAdam et al. [22]	USA	RCT	32 healthy male smokers and non-smokers aged 20–40 years	None	Coxib group 284 ± 107.2	220 ± 139.43	64 (−23.04,151.04)	pg/mg creatinine	None
					Placebo group 279 ± 103.1	218 ± 139.43	61 (−24.91, 146.91)		
Zedler et al. [24]	USA	Cross sectional	115 men and women at least 21 years	1 CPD + for the past year	All 1127.5 ± 290.7	715 ± 480.33	412.5 (270.63, 554.37)	pg/mg creatinine	None
Calapai et al. [10]	Italy	Cross sectional	60 healthy Caucasian men and women aged 23–43 years	Group B –intake below 60 mg tar/day; Group C –intake of more than 180 mg tar/day	All 1670 ± 660	1670 ± 660	460 (128.4, 791.6)	pg/mg creatinine	None
Frost-Pineda et al. ([36])	USA	Cross sectional	3346 adult smokers and 1051 non-smokers aged 21 or older. Men and women	1 CPD + for at least 1 year	All 1.34 ± 1.04	1.03 ± 0.79	0.31 (0.25, 0.37)	μg/24-h	None
Andreoli et al. [18]	Italy	Cross sectional	22 sets of healthy monozygotic twins discordant for smoking status. Men and women aged 23-46 years	Tar intake of ≥60 mg	All 3.02 ± 1.14	2.31 ± 0.78	0.71 (0.13, 1.29)	μg/24-h	None
Lowe et al. [14]	USA	Cross sectional	20 smokers and 20 non-smokers aged 21 + years	20 CPD + ISO yield from pack 10 mg tar	All 1.39 ± 0.81	1.09 ± 0.56	0.3 (0.26, 0.34)	μg/24-h	None
Haswell et al. [21]	Germany	Cohort	265 men and women aged 23-55 years.	10-30 CPD with an ISO tar yield of 6–8 mg.	All 0.8 ± 0.35	0.6 ± 0.34	0.20 (0.09-0.31)	Ug/24-h	None

CPD: Cigarettes per day, RCT: randomized controlled trial.

**Table 2:** Meta-Analysis results on smoking status and TXB2 levels.

Meta-Analyses	Studies (Estimates)^a^		Mean Difference Smokers – Non Smokers (95% CI)		
			Fixed Effects^b^	I^2^	Random Effects^b^
μg/24 h urine	3 [4]		0.30 (0.25, 0.35)	66%	0.33 (0.19, 0.48)
pg/mg creatinine	6 [7]		166.45 (120.51–212.40)	81%	230.87 (117.49, 344.26)

a The estimates used can be found in Table 1.

b The fixed effect model assumes that the estimated effects from the component studies in a meta-analysis come from a single homogeneous population, while the random effects model seeks to account for the fact that the study effect estimates are often more variable than assumed in the fixed effects model [38].

### 3.1 Effects of smoking status on urinary TXB2 levels

**Fig. 2:**
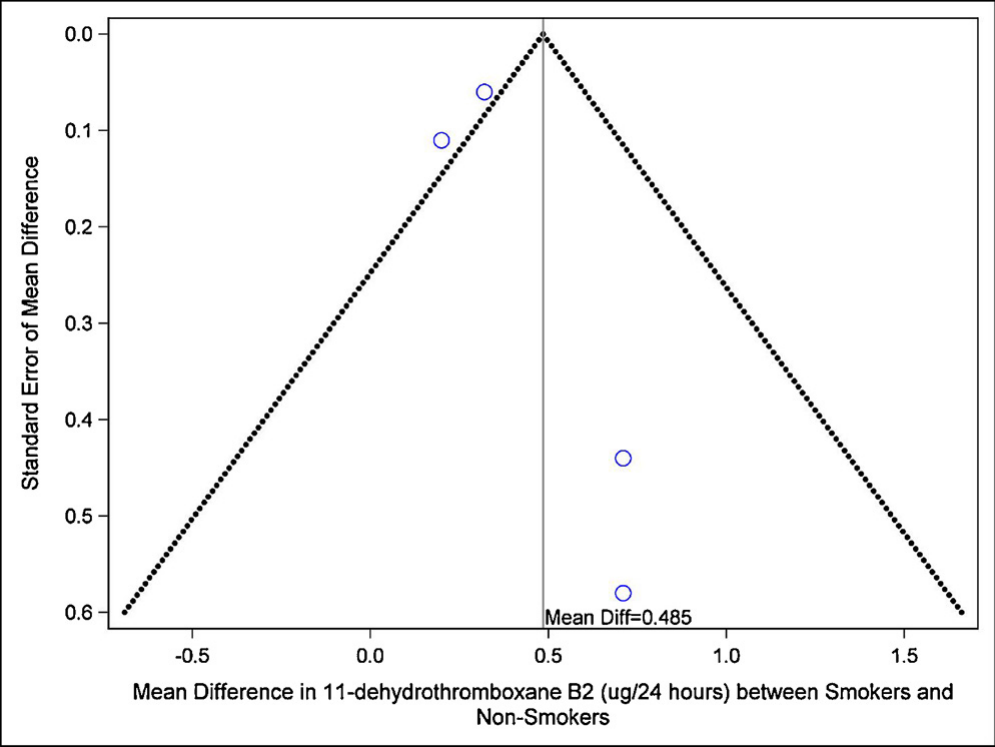
Funnel Plot of studies reporting excretion of TXB2 in 24-h urine (μg/24-h).

**Fig. 3:**
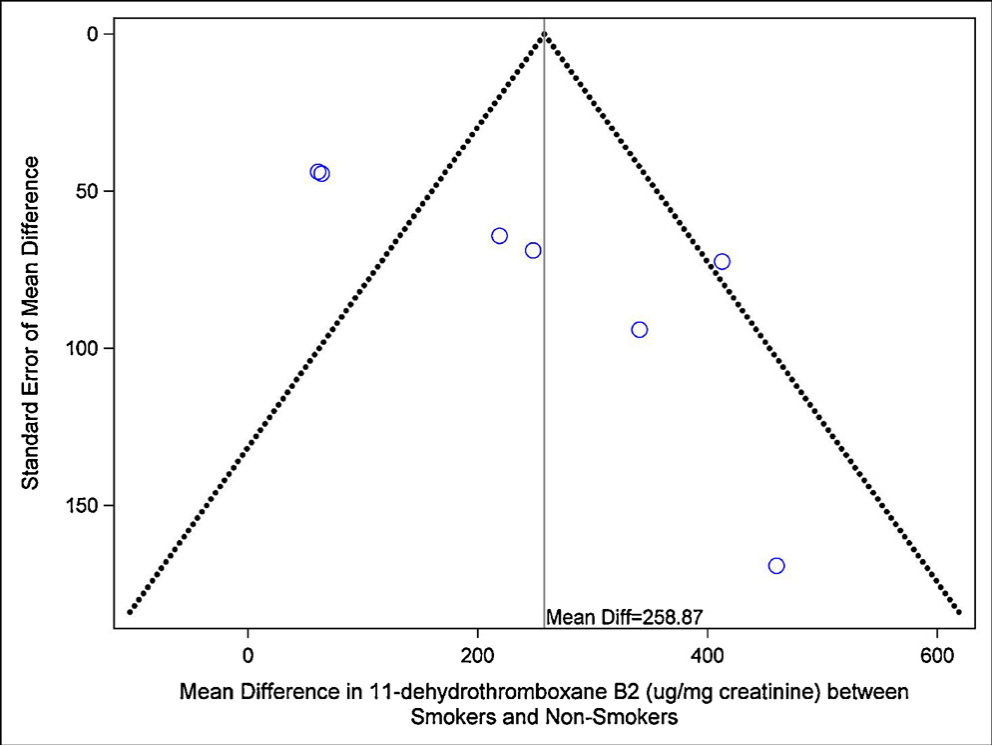
Funnel Plot of studies reporting excretion of TXB2 corrected for creatinine (pg/mg creatinine).

Of the 21 studies assessing the levels of TXB2 in smokers *vs*. non-smokers, ten reported data that could be used in the meta-analyses. The pooled-mean comparison analysis showed that excretion of TXB2 over 24 h urine was higher in smokers than non-smokers (pooled mean 532.62 μg/24-h in smokers *vs*. 366.2 μg/24-h in non-smokers). The same was found in the analysis of excretion adjusted to creatinine (1.12 pg/mg creatinine in smokers *vs*. 1.04 pg/mg creatinine in non-smokers). These results show an increase of 30–45% in TXB2 levels in smokers compared with non-smokers. The meta-analysis reporting 24-h urinary excretion of TXB2 (μg/24-h urine) included three studies [14,18,36] and showed statistically significantly increased levels of TXB2 in smokers *vs*. non-smokers as seen in Table 2 (Mean Difference = 0.30 pg/mg creatinine [95%CI: 0.27–0.34, p < 0.00001]). The inter-study heterogeneity in this meta-analysis was not statistically significant (I^2^ = 58%, p = 0.09) (Table 2). The meta-analysis of urinary TXB2 levels adjusted for creatinine excretion (μg/mg creatinine) included six studies which provided seven estimates. This analysis also found a statistically higher TXB2 level in smokers *vs*. non-smokers (Mean Difference = 166.45 μg/24-h [95%CI: 120.51–212.40, p < 0.00001]). In this comparison, however, the inter-study heterogeneity was statistically significant (I^2^ = 81%, p < 0.0001) (Table 2). Funnel plots are presented in Fig. 2 (for μg/24-h urine results) and Fig. 3 (for pg/mg creatinine). The evaluation of the funnel plots of both analyses does not point towards publication bias.

### 3.2 Effects of smoking cessation on urinary TXB2 levels

There were three studies assessing the effects of smoking cessation on urinary TXB2 levels [15,26,37]. All three studies found that smoking cessation reduced urinary TXB2 excretion. Urinary TXB2 levels decreased as early as three days after cessation [15,37]. The study by Rangemark et al. [37] reported a decrease to 60% of baseline values after 3 days of smoking abstinence, while the study by Benowitz et al. [26] reported a decrease to 78% of baseline values after five days of cessation, and the study by Saareks et al. [15] reported a decrease to 75% after three days and 50% after 14 days of cessation. Since the study by Saareks et al. [15] did not report actual values and the follow-up time was different in all studies, a meta-analysis could not be performed on the effect of cessation. The characteristics of the studies are found in Table 3.

**Table 3:** Characteristics of studies assessing levels of TXB2 after smoking cessation.

Study	Country	Study Design	Study Participants	Treatment	Findings
Benowitz et al. [26]	US	RCT	12 healthy male smokers	Participants went through three different phases, cigarette smoking, NRT and placebo NRT. Each treatment block lasted 5 days.	TXB2 levels were 611 ± 47 pg/mg creatinine in the period of CC smoking, 479 ± 34 pg/mg creatinine in the period of transdermal nicotine application and 496 ± 33 pg/mg creatinine during the placebo treatment.
Saareks et al. [15]	Finland	Cohort	60 men and women aged 20–45 years	Fifteen smokers quit smoking without nicotine substitution, 15 used nicotine chewing gum and 30 used nicotine patches as a substitution therapy	Three days after smoking cessation without nicotine substitution, 11-dehydrothromboxane B2 levels were lowered to 75% (*P* < 0.01) of the initial values, and after 14 days to 50% (*P* < 0.01).
Rangemark et al. [37]	Sweden	Cohort	8 women aged 23–45 years	No treatment	The basal excretion of 11-DTX-B2 was 586 ± 41 pg/mg creatinine (mean ± SEM), which fell to about 350 pg/mg after smoking cessation. The fall in excretion was complete after 3 days.

RCT: randomized controlled trial.

## 4 Discussion

The analyses showed that smokers had statistically significantly higher levels of urinary TXB2 in the pooled analyses. The 24-h excretion of urinary TXB2 in smokers was increased by 30% compared to non-smokers, while the amount of urinary TXB2 excreted adjusted for creatinine was increased by 45%. This is an important finding as levels of urinary TXB2 are associated with poorer prognosis in cardiovascular disease [[39], [40], [41], [42]].

There was substantial inter-study heterogeneity in the analysis of urinary TXB2 excreted adjusted for creatinine levels (I^2^ = 81%) however as proposed by von Hippel [43], when performing meta-analysis of only a few studies, the I^2^ values can be biased. In any case, the results of this meta-analysis should be interpreted with caution. There were not enough studies to perform meta-analyses on the effects of smoking cessation on urinary TXB2 levels, but all publications showed that cessation reduced levels of urinary TXB2, compared to baseline. Furthermore, the increased levels of TXB2 in smokers compared to non-smokers are in line with the levels seen at baseline in the smoking cessation studies where the TXB2 levels were up to 78% higher before quitting smoking [15].

Cigarette smoking is a strong risk factor for pulmonary as well as cardiovascular diseases [44]. Smoking cessation is the recommended method for avoiding such increased risk [14], but quitting has been proven difficult to achieve [45]. The FDA published draft guidelines on modified risk tobacco products (MRTPs) [46], which have led to the evaluation of risk reduction through the use of clinical risk markers [14], which should, in principle, be associated with smoking as well as be influenced by smoking cessation. One of the proposed clinical risk endpoints is thromboxane A_2_ [24]. Thromboxane A_2_ is produced by activated platelets, macrophages and neutrophils, it causes platelet aggregation and is a potent vasoconstrictor [47], hence it is a marker for platelet activation [11] and it has also been associated with a number of cardiovascular disorders [18]. Measurements of systemic thromboxane A2 have been proven difficult to test because of the complexity of measuring its production *in vivo*, with an extremely short half-life [19], nevertheless, other ways of assessing thromboxane production in a less invasive manner include the determination of its urinary metabolites, one of which is TXB2 [48]. In actuality, TXB2 measurements are used as a prognostic tool in acute myocardial infarction patients [40], for mortality in patients with stable coronary artery disease [39,41] and as a marker of vascular inflammation and prognostic in atherosclerotic cardiovascular disease [42].

The Cochrane Collaboration (www.chochrane.org) recommends meta-analyses as a statistical method to combine results of individual studies to allow researchers to make the best use of all available data and therefore increase the power of the analysis. As much as meta-analyses are a robust method, it has limitations, mainly concerning the identification of studies, inter-study heterogeneity and the availability of information [49]. For the smokers *vs*. non-smokers comparison there were 21 studies in the PubMed, SCOPUS and reference lists, but only ten had complete and useful information on TXB2 values that could be combined. For the effects of smoking cessation, only three studies were retrieved. One of the analyses of TXB2 levels between smokers and non-smokers (comparison in μg/24-h) showed no significant inter-study heterogeneity, thus facilitating the interpretation of results, while the second analysis (of studies reporting TXB2 levels in pg/mg creatinine) showed high inter-study heterogeneity, mainly due to the wide range of values reported per individual study. Additionally, there is always the possibility that a researcher’s bias could occur despite of the good design of the analyses. For this reason, two researchers performed the data extraction and discussed any discrepancies. Another potential limitation of this study is the possibility of publication bias [49], however, after evaluation of the funnel plots it does not seem that either analysis points towards publication bias. Finally, there is the possibility of confounding factors, as nine of the ten studies found in the comparison of urinary TXB2 levels in smokers *vs*. non-smokers were observational in design and the mean values reported were not adjusted for possible confounding variables. However, the fact that the three studies evaluating the effect of smoking cessation on urinary TXB2 levels show a marked decrease after quitting [37], suggests that there is indeed an effect of smoking in urinary TBX2 levels.

## 5 Conclusion

This meta-analyses show that urinary TXB2 is a clinical risk endpoint that is significantly increased in cigarette smokers compared to non-smokers. The reviewed data further indicate that urinary TXB2 levels are reversible as early as three to five days after smoking cessation as TXB2 levels decrease after quitting, although longer follow-up research is needed to understand whether the level of change after cessation could impact clinical disease outcomes. These data suggest that urinary TXB2 could be a potential candidate endpoint with which to assess risk reduction of candidate MRTPs.

## Transparency document

Transparency Document

## Footnotes

1 TXB2= 11-dehydrothromboxane_b2_.
